# Dengue fever mimicking acute appendicitis: A case report^☆^

**DOI:** 10.1016/j.ijscr.2013.08.017

**Published:** 2013-09-08

**Authors:** M.E.C. Mcfarlane, J.M. Plummer, P.A. Leake, L. Powell, V. Chand, S. Chung, K. Tulloch

**Affiliations:** Department of Surgery, Radiology, Anaesthetics and Intensive Care, University of the West Indies, Mona, Jamaica

**Keywords:** Dengue fever, Acute appendicitis, Acute abdomen, Leucopenia

## Abstract

**INTRODUCTION:**

Dengue fever is an acute viral disease, which usually presents as a mild febrile illness. Patients with severe disease present with dengue haemorrhagic fever or dengue toxic shock syndrome. Rarely, it presents with abdominal symptoms mimicking acute appendicitis. We present a case of a male patient presenting with right iliac fossa pain and suspected acute appendicitis that was later diagnosed with dengue fever following a negative appendicectomy.

**PRESENTATION OF CASE:**

A 13-year old male patient presented with fever, localized right-sided abdominal pain and vomiting. Abdominal ultrasound was not helpful and appendicectomy was performed due to worsening abdominal signs and an elevated temperature. A normal appendix with enlarged mesenteric nodes was found at surgery. Complete blood count showed thrombocytopenia with leucopenia. Dengue fever was now suspected and confirmed by IgM enzyme-linked immunosorbent assay against dengue virus.

**DISCUSSION:**

This unusual presentation of dengue fever mimicking acute appendicitis should be suspected during viral outbreaks and in patients with atypical symptoms and cytopenias on blood evaluation in order to prevent unnecessary surgery.

**CONCLUSION:**

This case highlights the occurrence of abdominal symptoms and complications that may accompany dengue fever. Early recognition of dengue fever mimicking acute appendicitis will avoid non-therapeutic operation and the diagnosis may be aided by blood investigations indicating a leucopenia, which is uncommon in patients with suppurative acute appendicitis.

## 1 Introduction

Dengue fever is the most common vector-borne viral illness worldwide with frequent outbreaks and an estimated 100 million cases worldwide.^1^ In Jamaica it is a frequent cause of acute febrile illness, and is one of the most common mosquito borne diseases seen most often during the rainy season when the Aedes agaepti mosquito breed.^2^ The clinical symptoms of dengue usually appear 3–4 days after exposure to the virus and include high fever and a rash, with headaches, joint and muscle pains, nausea and vomiting.^3^ Though generally a mild self-limiting disease, severe complications have been described including dengue haemorrhagic fever and dengue shock syndrome. There have also been reports of unusual clinical presentations of patients with myocarditis,^4^ dengue encephalitis,^5^ liver failure and gastrointestinal haemorrhage.^6^ There are few reports of surgical emergencies occurring during dengue fever such as acalculous cholecystitis, acute pancreatitis, and symptoms mimicking acute appendicitis. Prompt recognition of the features of dengue fever in patients with signs mimicking acute appendicitis will avoid the morbidity of non-therapeutic appendicectomy. We report a patient who presented with right-sided abdominal pain typical of acute appendicitis that was later confirmed as dengue fever.

## 2 Case report

A 13-year old male with no previous medical history presented to the surgical unit of the University Hospital of the West Indies with a 3-day history of abdominal pain that began in the epigastrium but later became localized in the right iliac fossa. The pain was associated with several bouts of vomiting. There was no diarrhoea, joint pain, or petechial rash.

Physical examination revealed a temperature of 38 °C, pulse rate 109 min^−1^ and normal blood pressure. Examination of the abdomen revealed marked tenderness in the right iliac fossa with mild guarding.

Complete blood count revealed thrombocytopenia (platelet count 119 × 10^6^ mm^−3^) and leucopenia (white blood cell count of 2.1 × 10^6^ mm^−3^). Haemoglobin and haematocrit were normal. An abdominal ultrasound was performed and showed a small fluid collection in the right iliac fossa, however the appendix was not identified. There was normal peristalsis of bowel loops.

The patient was observed for 24 h in hospital and was noted to have worsening right iliac fossa pain and leucopenia on the second day. Examination at that time revealed marked right iliac fossa tenderness in keeping with a diagnosis of acute appendicitis. Because the signs were thought to be typical of appendicitis diagnostic laparoscopy was not deemed necessary and an appendicectomy performed via a Lanz incision, at which time a normal appendix was found with multiple enlarged mesenteric lymph nodes. Lymph node biopsy was not done.

Repeat blood investigations again showed bicytopenia with platelet count of 99 × 10^6^ mm^−3^ and a white cell count of 2.3 × 10^6^ mm^−3^.

Dengue fever was suspected and was confirmed with a positive IgG and IgM enzyme-linked immmunoabsorbent assay.

The histology of the appendix showed no gross abnormality. The patient received supportive care, which included analgesics, bed rest and fluid therapy and made an uneventful recovery. Repeat blood investigations one week later were normal.

The patient was seen one year later in the outpatients department at which time blood results were repeated and were normal.

## 3 Discussion

Dengue fever is recognized as the most common flavivirus infection worldwide with an estimated prevalence of 50–100 million cases annually with nearly 500,000 cases of dengue haemorrhagic fever.^1^ The disease is common in most tropical countries including Jamaica with outbreaks usually limited to the rainy season.^2,7^ The virus is found as four closely related serotyopes (DEN1–DEN4) and is transmitted between humans by the two species of mosquitos namely the Aedes aegypti and Aedes albopictus.^8,9^

Dengue fever usually presents as an acute febrile illness, retroorbital pain, muscle and joint pains, nausea, vomiting, and a petechial rash. Severe disease manifests as dengue haemorrhagic fever or dengue shock syndrome. Abdominal pains with features of the acute abdomen have been rarely reported. These cases pose difficulties with diagnosis and management because of the non-specific presentation. The spectrum of acute surgical emergencies which raise suspicion of an abdominal catastrophe in patients presenting with dengue fever include, acute pancreatitis,^10^ acute acalculous cholecystitis,^11,12^ non-specific peritonitis and acute appendicitis.^13^

Premaratna et al. reported 12 cases of dengue fever mimicking acute appendicitis. All 12 patients presented with right iliac fossa pain with severe tenderness and with eight of twelve patients having leucopenia. Ten out of twelve patients had thrombocytopenia on admission with all patients developing a low platelet count at the time of discharge.

The patient in this case report presented with leucopenia and thrombocytopenia, which worsened during the course of admission. The presence of low white cell count and platelet count though not diagnostic of dengue fever, can raise suspicion of a diagnosis of dengue in a patient presenting with acute abdominal pain, during a dengue epidemic.

In a recent review of 357 patients with dengue fever, 276 had nonspecific abdominal pain without overt abdominal signs, 43 (12.04%) had acute abdominal pain with definite abdominal signs and only 38 (10.64%) presented without abdominal pain.^14^

The incidence of acute abdominal signs reported in patients with dengue fever has ranged from 4.3%^12^ to 12.04%.^14^ The male:female ratio for patients presenting with dengue fever is nearly 1:1, but the few reports of patients presenting with acute abdomen showed that females were more frequently affected than males.^12,14^

The cause of the onset of severe abdominal pain associated with signs of the acute abdomen in patients with dengue fever is unclear. The histological finding of enlarged mesenteric lymph nodes with serous fluid collection and oedema may result in the inflammatory changes identified in patients with acalculous cholecystitis and acute appendicitis. Spasms of the cystic duct, gall bladder distension and cholestasis have been suggested as possible causes of acalculous cholecystitis. Enlarged mesenteric lymph nodes as was found in our patient may explain the acute right iliac fossa pain mimicking acute appendicitis similar to the presentation in patients with mesenteric adenitis. Reviews of the literature have failed to identify any specific aetiological agent or features on pathological analysis that can account for this presentation.^15^

In a series of patient reported by Shamim et al.^14^ all patients with acute abdominal pain had complications of dengue fever namely dengue haemorrrhagic fever or dengue shock syndrome. Other theories proposed to explain acute abdominal pain in patients with dengue fever include plasma leakage and serious effusions containing high protein content together with lymphocytic infiltrations in patients presenting with acute appendicitis, pancreatitis and acalculous cholecystitis.^12,15^

## 4 Conclusions

Dengue fever though presenting as a febrile viral illness, rarely presents as an acute abdominal emergency mimicking acute appendicitis. Nevertheless this presentation should be suspected in patients with cytopenia and abdominal pain particularly during a viral outbreak of dengue fever and prompt the early use of serological assays. This awareness should be heightened in tropical and endemic geographical regions.

## Conflict of interest

None.

## Funding

None.

## Ethical approval

Informed consent obtained.

## Author contributions

All authors contributed to paper.

## References

[1] Pan American Health Organisation (2002). Workshop on dengue burden studies.

[2] Brown M.G., Salas R.A., Vickers I.E., Heslop O.D., Smikle M.F. (2011). Dengue virus serotypes in Jamaica, 2003–2007. West Indian Medical Journal.

[3] Kyle J.L., Harris E. (2008). Global spread and persistence of dengue. Annual Review of Microbiology.

[4] Promphan W., Sopontammarak S., Pruekprasert P., Kajornwattanakul W., Kongpattanayothin A. (2004). Dengue myocarditis. Southeast Asian Journal of Tropical Medicine and Public Health.

[5] Muzaffar J., Venkata Krishnan P., Gupta N., Kar P. (2006). Dengue encephalitis: why we need to identify this entity in a dengue-prone region. Singapore Medical Journal.

[6] Vinodh B.N., Bammigatti C., Kumar A., Mittal V. (2005). Dengue fever with acute liver failure. Journal of Postgraduate Medicine.

[7] Shuaib F., Todd D., Campbell-Stennett D., Ehiri J., Jolly P.E. (2010). Knowledge, attitudes and practices regarding dengue infection in Westmoreland, Jamaica. West Indian Medical Journal.

[8] Singhi S., Kissoon N., Bansal A. (2007). Dengue and dengue hemorrhagic fever: management issues in an intensive care unit. Journal of Pediatrics.

[9] Brown M.G., Salas R.A., Vickers I.E., Heslop O.D., Smikle M.F. (2011). Molecular epidemiology of dengue in Jamaica dengue virus genotypes in Jamaica, 2007. West Indian Medical Journal.

[10] Derycke T., Levy P., Genelle B., Ruszniewski P., Merzeau C. (2005). Acute pancreatitis secondary to dengue. Gastroenterologie Clinique et Biologique.

[11] Goh B.K., Tan S.G. (2006). Case of dengue virus infection presenting with acute acalculous cholecystitis. Journal of Gastroenterology and Hepatology.

[12] Khor B.S., Liu J.W., Lee I.K., Yang K.D. (2006). Dengue hemorrhagic fever patients with acute abdomen: clinical experience of 14 cases. American Journal of Tropical Medicine and Hygiene.

[13] Premaratna R., Bailey M.S., Ratnasena B.G., de Silva H.J. (2007). Dengue fever mimicking acute appendicitis. Transactions of the Royal Society of Tropical Medicine and Hygiene.

[14] Shamim M. (2010). Frequency, pattern and management of acute abdomen in dengue fever in Karachi, Pakistan. Asian Journal of Surgery.

[15] Wu K.L., Changchien C.S., Kuo C.M., Chuah S.K., Lu S.N., Eng H.L., Kuo C.H. (2003). Dengue fever with acute acalculous cholecystitis. American Journal of Tropical Medicine and Hygiene.

